# ‘Preparing to prescribe’: an online implementation toolkit for non-medical prescribers

**DOI:** 10.29045/14784726.2021.3.5.4.59

**Published:** 2021-03-01

**Authors:** Karen Stenner, Nicola Carey

**Affiliations:** University of Surrey; University of Surrey

The introduction of prescribing for advanced paramedics signals a step up in the expansion of paramedic practice into new and emerging clinical roles. Many benefits are anticipated from paramedic prescribing, including improved access and quality of care for patients and enhanced service efficiency (NHSE, 2015). However, barriers can also present at different stages of the prescribing journey to prevent optimal use of the prescribing qualification (Noblet et al., 2017). Having realistic expectations of the future prescribing role and a solid understanding of course requirements is essential for both individuals and organisations. This preparation is particularly important as, rather than starting with supplementary prescribing, paramedics are the first profession to be awarded joint independent/supplementary prescribing responsibilities.

We are delighted to introduce the updated version of the ‘preparing to prescribe’ toolkit which may be of value to those considering undertaking the prescribing programme: https://www.surreytoolkit.uk/toolkit-main/.

‘Preparing to prescribe’ is a Surrey Implementation toolkit™ that ensures those who are interested in undertaking prescribing training receive consistent pre-course information, and provides resources for healthcare professionals, non-medical prescribing leads, service and provider organisations, commissioners and universities to support implementation of non-medical prescribing in practice.

The ‘preparing to prescribe’ toolkit consists of trigger questions, signposting and links to current guidance and is designed to support those who wish to become a non-medical prescriber. By working with their organisation and making appropriate plans, those who undertake non-medical prescribing training will be better placed to ensure effective implementation of the prescribing role in practice.

Paramedic prescribing is likely to become increasingly important given predicted workforce deficits. ‘Preparing to prescribe’ can help optimise the readiness of individuals and organisations, and to realise the full potential of the prescribing role.

If you have any suggestions for further improvements, please contact Dr Nicola Carey at n.carey@surrey.ac.uk or Dr Karen Stenner at k.stenner@surrey.ac.uk.

Access the toolkit at: https://www.surreytoolkit.uk/toolkit-main/.
